# Contributed by One of Them

**Published:** 1920-10-30

**Authors:** 


### THE POSITION OF WOMEN AT CAMBRIDGE.

### Contributed by One of Them.

The University of Cambridge is face to face with a problem of the utmost importance. She stands alone among the Universities of the British Isles in not admitting women to membership of the University. In 1897 the Senate rejected the proposals of a Syndicate to admit women to degrees, and to-day they are facing the question again, and have been discussing the proposals of a Syndicate appointed in December 1919 to consider the relation of women to the University. Oxford, the sister University, has settled the question easily, apparently regarding the admission of women to full membership on the same terms as the men as an inevitable outcome of present day conditions. Cambridge, on the other hand, regards the question as involving fundamental issues; and much public interest has been aroused by the discussion of the different opinions which have been put forward, based upon the divergent reports issued by the Syndicate appointed to consider the matter.

### Reports of Syndicate.

Two reports were presented in May 1920, the members of Syndicate being divided in the opinion upon the question, and consequently making different recommendations. The Syndicate was appointed to consider "whether women students should be admitted to the University, and if so with what limitations, if any; and alternatively to consider if women students are not admitted to membership, by what means the University could co-operate with the Women's Colleges or other bodies in the conferment of degrees on women students.

The two reports have an equal number of signatories, so that there is no question of a majority and minority report. Report A recommends admission of women to full membership of the University, with certain limitations calculated to prevent their admission to men's colleges. Posts and emoluments carrying with them conditions of residence in men's colleges could therefore not be open to women. Further, according to the proposed statute women should only be admitted for matriculation or degrees from a women's college, or public hostel for women recognised by the University. Non-collegiate women students are therefore not admitted, and some control over numbers rests with the University in that it must give its sanction and recognition to new colleges or hostels. This recommendation meets with the full approval of women, who have expressly stated that they have no desire to enter men's colleges both in the Press and in the memorial in support of Report A, which has been presented to the Senate and signed by 2,500 past students of Girton and Newnham Colleges.

The members of the Syndicate who opposed this recommendation do so upon two main grounds. In the first place, they fear that the restrictions imposed, though now meeting with acquiescence, will prove in the future a source of grievance, and that women will be demanding entrance to men's colleges and urging the rights of non-collegiate students—this objection is one which affects the University and its administration, and could, one would think, be overcome by statutory enactment. There are difficulties, certainly, in that the colleges are to a great extent self-governing, and might individually appoint women to their staff; but the several questions raised are matters affecting the constitution of the University, and as such do not affect the nation as a whole. The other objection raised by the supporters of Report B seems to us to be one of fundamental importance, in that it lays stress on the distinction of sex in educational matters generally. It is questioned whether the University education which is suitable for men is also suitable for women, and Report B advocates the establishment of a separate University for women.

### Main Question at Issue.

There are many points arising out of the controversy, such as overcrowding, discipline, offices which might or might not be held by women, all of which invite discussion, but all of which seem to be subsidiary to the main question:- Is it desirable in the interests of the nation that women should be given the same education as men at the University, and upon the same footing? Is there to be sex-distinction in education? The question is a fundamental one, especially in view of the position of women in public affairs to-day, and it is a question which is evoking much interest. Supporters of the idea of a separate University have put forward the view that what women need is not an education of a higher or lower kind, but of a "different" kind. Women who have worked in connection with Cambridge feel that their work for the past fifty years has proved that they are worthy to become part of the University to which they owe so much. Their intellectual attainments have not been insignificant, as the names of Miss Ramsay, Miss Fawcett and others go to prove. They are strongly against the idea of a separate University for women, and it must be remembered that these past Cambridge students are women holding high places in the educational and public life of the country whose opinion cannot be ignored.

A lowering in the standard of educational ideals of the nation is feared by the opponents of the admission of women, if in every University in the kingdom there is a mixture of men and women.

### Present-Day Conditions.

It must be admitted by all, however, that in the public life of the nation, especially since the passing of the Sex Disqualification Bill, men and women tend more and more to meet on an equal footing. In the professions, in Parliament, in public social work and in educational work women play a part, in some cases a large part, and it is difficult to see wherein educational preparation for these callings should differ for men and women. The questions raised by Cambridge are fundamental and it is right that they should be discussed openly and with a view to a settlement which will be of benefit to men and women alike. At the same time it is felt that co-operation in University life between men and women cannot but make for co-operation later in public life, and it is mutual understanding between individuals and classes that is so essential in our national life to-day.

